# The effect of water and shampooing on the efficacy of fluralaner spot-on solution against *Ixodes ricinus* and *Ctenocephalides felis* infestations in dogs

**DOI:** 10.1186/s13071-016-1367-y

**Published:** 2016-05-31

**Authors:** Janina Taenzler, Boyd Gale, Eva Zschiesche, Rainer K. A. Roepke, Anja R. Heckeroth

**Affiliations:** MSD Animal Health Innovation GmbH, Zur Propstei, 55270 Schwabenheim, Germany; Charles River Laboratories Preclinical Services Ireland Ltd, Glenamoy, Co. Mayo, Ireland

**Keywords:** Bathing, Bravecto™ spot-on solution, Fluralaner, Dog, Flea, *Ctenocephalides felis*, Efficacy, Water-immersion, Shampooing, Tick, *Ixodes ricinus*

## Abstract

**Background:**

Fluralaner spot-on solution provides immediate and persistent efficacy against tick and flea infestations in dogs and cats for 12-weeks following topical administration. The active ingredient fluralaner is distributed systemically following transdermal absorption. Therefore, this study tested the hypothesis whether water-immersion or shampooing of dogs following administration of fluralaner spot-on solution has an impact on subsequent tick and flea efficacy.

**Methods:**

Thirty-two Beagle dogs were allocated to four study groups of 8 dogs each. On day 0, dogs in the 2 treatment groups received topical administration of fluralaner (Bravecto™ spot-on solution) according to label instructions. Dogs in the 2 corresponding control groups remained untreated. On days 3, 21, 49, and 77 dogs in one treatment group and control group were water-immersed for 2–5 min, while dogs in the other treatment group and control group were shampooed 6–8 min with a commercial foaming micro-emulsion, unscented product. On days 4, 28, 56, and 84 all dogs were co-infested with 50 ± 2 female and 10 ± 2 male *Ixodes ricinus* and 100 ± 4 *Ctenocephalides felis,* with tick and flea removal and counts 48 ± 2 h post-infestation. Efficacy against ticks and fleas was calculated for each assessment time point.

**Results:**

No treatment-related adverse event was observed in any of the 16 dogs treated with fluralaner spot-on solution during the study.

Efficacy against ticks at each assessment time point was between 99.7 and 100 % in the water-immersed group and between 99.2 and 100 % in the shampooed group. Efficacy against fleas was 100 % at each assessment time point as well in the water-immersed as the shampooed group. Tick and flea reduction in both treatment groups was significant at all assessment time points (*p* < 0.0001).

**Conclusions:**

Neither water-immersion nor shampooing after single topical administration of fluralaner spot-on solution had an impact on the excellent tick and flea efficacy over the 12-week recommended re-treatment interval.

## Background

Ticks and fleas are the most important ectoparasites infesting dogs worldwide. They cause discomfort and blood loss for infested dogs and also create concerns for owners when their pets are infested. Furthermore, there is risk for secondary complications such as flea allergy dermatitis (FAD) [1], and transmission of pathogens via blood-feeding parasites [2, 3]. Thus, tick and flea control for dogs continues to be of great medical and veterinary importance.

During the last decade the number of products and strategies available for tick and flea control increased remarkably, e.g. by combining several active ingredients into one formulation to broaden the anti-parasitic spectrum, or presenting different routes of administration, including oral, injectable and topical administration, giving the veterinarian and pet owner the option of choosing their preferred product [4, 5].

Products with a topical route of administration can potentially have their duration and level of efficacy affected by water (i.e. rainfall or swimming) or owner practices (i.e. shampooing of the dog). For example, some dogs (e.g. those with an outdoor or working lifestyle) may get a lot of water exposure and/or owners may frequently bathe their dog for cosmetic or health reasons. Water exposure can markedly shorten the period of flea effectiveness of imidacloprid and flumethrin administered topically in a collar presentation [6]. Therefore, for a topically administered ectoparasiticide it is important to ensure that the treatment maintains its effectiveness over the entire recommended treatment interval including the possibility of repeated water-immersion or shampooing.

Bravecto™, with its active ingredient fluralaner, is commercially available as a flavoured chewable tablet [7] and also more recently as a topical solution [8]. In both formulations, fluralaner is distributed systemically and ectoparasites are exposed through feeding activity. Both chewable and topical presentations have demonstrated an efficacy duration of 12 weeks and provide immediate and persistent killing of ticks and fleas on dogs. As fluralaner spot-on solution is administered topically and distributed systemically following transdermal absorption, it is hypothesized that bathing or shampooing a treated dog will have no impact on the subsequent tick and flea killing efficacy. Thus, the aim of this study was to evaluate this hypothesis by assessing the potential effect of water-immersion (simulating swimming of the dog) and the potential effect of shampooing on the persistent tick and flea killing efficacy after a single administration of fluralaner spot-on solution to dogs.

## Methods

### Study set-up

The study was conducted in accordance with Good Clinical Practice (VICH guideline GL9, Good Clinical Practice, EMA, 2000), and was in compliance with the Statutory Instrument S.I. No. 566 of 2002, which incorporates EC directive, 86/609/EC into Irish law. The study was submitted to the Ethics Committee of Charles River Laboratories Preclinical Services Ireland Ltd that authorized the conduct of the study. The study was performed as a negative controlled, blinded, randomized efficacy study.

In total, 32 Beagles (16 males, 16 females) with an intact hair coat at the administration site (i.e., dorsal back region), over 6 months of age and weighing between 8.1 and 18.9 kg were included. Each dog was in good health, had not been treated with any parasite control product within 3 months prior to an 8 day acclimatization period, and was uniquely identified by a microchip number.

Prior to randomization, dogs were clinically examined and the parasite susceptibility of each dog was confirmed by a flea infestation with 100 (±4) unfed adult *Ctenocephalides felis* fleas followed by flea removal and count 48 h (±2 h) later. The flea isolate used in the study originated from the University of Cardiff, United Kingdom, and has been maintained since 1996, with the last outside introduction in August 2004, at Charles River Laboratories Preclinical Services Ireland Ltd. All dogs included in the study harboured more than 50 % of the number of originally infested fleas. Ranking of the dogs was performed within gender by descending flea counts and dogs were randomly allocated to 4 study groups (2 treatment and 2 control groups, each for water immersion or shampooing) of 8 dogs each using a computer generated randomization list.

All dogs were kept indoors. During periods without parasite infestation, dogs were group-housed within their corresponding study group, while during periods of parasite infestations, all dogs were housed individually. On days of individual housing and without any activities that involved interaction with the dog, each dog had social interaction for a minimum of 2 min. Social interaction included either rubbing, talking to or playing with the animal in its pen.

Temperature in the dog housing facility ranged between 16 and 20 °C and the relative humidity between 38 and 73 %. Dogs were fed a standard commercially available dry dog food once daily, and drinking water was provided *ad libitum*. General health observations were performed once daily throughout the study.

### Treatment

On day 0 (i.e., day of treatment), dogs in the 2 treatment groups were treated once topically with fluralaner (Bravecto™ spot-on solution) according to the manufacturer’s label instructions. Before administration, the skin and hair of the designated administration sites were inspected visually for any abnormalities, which were not observed. Fluralaner spot-on solution was administered topically as one or two spots along the dog’s dorsal line depending on the administration volume. The first spot was administered between the shoulder blades and the second spot approximately 5–10 cm caudal of the previous spot. The hair was parted and the tip of the pipette was placed vertically on the skin and the solution administered directly to the skin by pressing the corpus of the pipette to empty its contents. After administration, the dog was held upright for approximately 5 min to determine, if any of the solution ran off the animal during or directly (up to 5 ± 2 min) after treatment. There was no evidence of mis-dosing such as spillage or run-off/drip-off in any treated animal. Dogs in both control groups remained untreated.

### Water-immersion and shampooing of dogs

On days 3, 21, 49, and 77 after treatment, dogs of one treatment group were water-immersed and dogs of the other treatment group were shampooed using a locally procured commercial foaming micro-emulsion and unscented shampoo without any insecticide for dogs (Allermyl® shampoo, Virbac Animal Health, France). Dogs of the corresponding control group were either water-immersed or shampooed, respectively, at the same defined times after treatment as dogs in the corresponding treatment group. For water-immersion, each dog was water immersed for 2 – 5 min in a separate tank of fresh lukewarm water while holding the head of the dog out of the water. The head was wetted by pouring water over it. For shampooing (lasted 6–8 min), the entire body of each dog was first wetted with lukewarm water. Then 15–20 mL of shampoo was applied to the entire body (avoiding the nose and eyes region). The dog’s hair coat was massaged until foam formation. Thereafter, the dog was rinsed thoroughly with fresh lukewarm water to remove all residual shampoo. After water-immersion or shampooing, each dog was dried with a separate towel and then air dried.

### Co-infestations with ticks and fleas and their assessments

The tick isolate used in the study originated from Slovakia, Germany and Ireland, and has been maintained since 2004, with annual refreshments, at Charles River Laboratories Preclinical Services Ireland Ltd. Tick and flea co-infestations were conducted on ketamine/xylazine sedated dogs on days 4, 28 (4 weeks), 56 (8 weeks), and 84 (12 weeks) after treatment. At each infestation time point each dog was co-infested with 100 (±4) unfed adult *C. felis* fleas and 60 (±4) viable unfed adult *I. ricinus* ticks (50 ± 2 females and 10 ± 2 males). The 10 male ticks were additionally applied to give *Ixodes* females best conditions for attachment. Tick and flea removal and counts were performed 48 h (±2 h) after each infestation. Ticks were classified as dead or alive, attached or unattached, and engorged or unengorged. Fleas were classified as dead or alive and only live adult fleas were counted. The personnel conducting tick/flea classification and tick/flea counts were blinded to the treatment status of each dog.

### Efficacy evaluation

The statistical analysis was performed using the software package SAS® (SAS Institute Inc., Cary, NC, USA, release 9.2). The individual dog was the experimental unit in all statistical calculations. Data from each tick and flea count time point were analysed separately.

The percentage of efficacy against ticks and fleas was calculated for each treatment group at each assessment time point using geometric means with Abbott’s formula:

Efficacy (%) = 100 x (M_C_ - M_T_) / M_C,_ where M_C_ was the mean number of total live female ticks (attached and free) or total adult live fleas on untreated control dogs (water-immersed or shampooed group) and M_T_ the mean number of total live female ticks (attached and free) or total adult live fleas on treated dogs (water-immersed or shampooed group). In case of zero counts, the geometric mean (of ticks or fleas) was calculated as follows:

$$ {\mathrm{x}}_{\mathrm{g}}={\left({\displaystyle \prod_{\mathrm{i}=1}^{\mathrm{n}}\left({\mathrm{x}}_{\mathrm{i}}+1\right)}\right)}^{\frac{1}{\mathrm{n}}}-1 $$

Significant differences were assessed between the log-counts of live ticks and fleas in each treatment group at each assessment time point and the log-counts of the corresponding untreated control group. Study groups were compared using a linear mixed model including study group as a fixed effect and block as a random effect. The two-sided level of significance was declared when *p* ≤ 0.05.

## Results

No treatment-related adverse event was observed in any of the 16 dogs treated with fluralaner spot-on solution during the study.

An adequate tick infestation (at least 6 out of 8 animals with a tick attachment rate > 25 %) was achieved in both control groups on all infestation time points, except in the shampooed control animals on day 58. At this time point, 5 of the 8 control animals had a tick attachment rate >25 %, and the individual tick attachment rate ranged between 5.0 and 51.5 %. However, as this only occurred on one occasion and involved only one dog, the tick infestations were considered to be sufficiently vigorous to provide a valid result. The efficacy against ticks at each assessment time point was 99.7-100 % after a single topical fluralaner spot-on solution administration in the water-immersed group. In the shampooed group, the efficacy against ticks at each assessment time point was 99.2-100 % after a single topical fluralaner spot-on solution treatment. Tick reduction in both treatment groups was significant at all assessment time points (*p* < 0.0001) (Table 1).

**Table 1:** Mean tick counts and efficacy (%) after a single topical administration of fluralaner spot-on solution followed by water-immersion or shampooing of dogs before each tick infestation

Assessment time points^a^		Study day 6		Study day 30		Study day 58		Study day 86	
Study Group		Fluralaner	Control	Fluralaner	Control	Fluralaner	Control	Fluralaner	Control
Water-immersion	Mean^b^ tick counts (n)	0.0	27.5	0.0	29.7	0.0	19.3	0.1	28.7
	Count range (n)	0	18–38	0	21–35	0	9^d^–39	0–1	21–32
	Efficacy (%)	100^c^		100^c^		100^c^		99.7^c^	
Shampooing	Mean^b^ tick counts (n)	0.0	28.9	0.0	28.5	0.0	14.3	0.2	29.6
	Count range (n)	0	20–39	0	20–37	0	3^e^–31	0–4	21–41
	Efficacy (%)	100^c^		100^c^		100^c^		99.2^c^	

^a^Assessment of ticks 48 h (±2 h) after treatment or re-infestation following treatment

^b^Geometric mean

^c^Log-counts of live ticks from the treatment group were significantly different (*p* ≤ 0.05) from log-counts of the respective untreated control group

^d^7 out of 8 dogs adequately infested

^e^5 out of 8 dogs adequately infested

An adequate flea infestation (at least 6 out of 8 animals with a flea infestation rate > 50 %) was achieved in both control groups on all infestation time points, except in the shampooed control animals on day 6. At this time point, 4 of the 8 control animals had a flea infestation rate >50 %, and the individual flea infestation rate ranged from 6 to 97 %. The lower infestation rate in these dogs may have been caused by the dogs being shampooed 1 day before the flea infestation on day 4. At all other infestation time points the dogs were shampooed 7 days before flea infestation. However, as this only occurred on one occasion, the flea infestations were considered to be sufficiently vigorous to provide a valid result. The efficacy against fleas at each assessment time point was 100 % after a single topical fluralaner spot-on solution administration in the water-immersed and in the shampooed group. Flea reduction in both treatment groups was significant at all assessment time points (*p* < 0.0001) (Table 2).

**Table 2:** Mean flea counts and efficacy (%) after a single topical administration of fluralaner spot-on solution followed by water-immersion or shampooing of dogs before each flea infestation

Assessment time points^a^		Study day 6		Study day 30		Study day 58		Study day 86	
Study Group		Fluralaner	Control	Fluralaner	Control	Fluralaner	Control	Fluralaner	Control
Water-immersion	Mean^b^ flea counts (n)	0.0	92.2	0.0	88.6	0.0	88.3	0.0	81.7
	Count range (n)	0	85–99	0	78–101	0	79–100	0	65–96
	Efficacy (%)	100^c^		100^c^		100^c^		100^c^	
Shampooing	Mean^b^ flea counts (n)	0.0	34.5	0.0	80.2	0.0	87.7	0.0	89.1
	Count range (n)	0	6^d^–97	0	55–101	0	78–101	0	73–99
	Efficacy (%)	100^c^		100^c^		100^c^		100^c^	

^a^Assessment for fleas 48 h (±2 h) after treatment or re-infestation following treatment

^b^Geometric mean

^c^Log-counts of live fleas from the treatment group were significantly different (*p* ≤ 0.05) from log-counts of the respective untreated control group

^d^4 out of 8 dogs adequately infested

## Discussion

Results from this study demonstrate that a single topical fluralaner spot-on solution administration remains highly effective against ticks and fleas for 12 weeks following treatment even in the face of repeated water-immersion (simulating swimming of the dog) or shampooing of the dog. Coat wetting is inevitable for dogs, and dogs that spend a lot of time outdoors are likely to have an increased level of water exposure including swimming and rainfall. Additionally, bathing and shampooing dogs for cosmetic and hygienic reasons is a common practice. Therefore, it is important that a topically applied, long-acting ectoparasiticide retains its efficacy over the complete re-treatment interval even in the face of water-immersion or shampooing. This study showed that either soaking with water or shampooing the dog with a foaming micro-emulsion and unscented shampoo without any insecticide up to a total of 4 times within 12 weeks, had no impact on the duration and level of efficacy of fluralaner spot-on solution over the 12-week recommended re-treatment interval. Therefore, veterinarians and pet owners do not need to be concerned that tick and flea efficacy will diminish, if a dog treated with fluralaner spot-on solution goes swimming, gets exposed to rain or will be shampooed.

The first water-immersion or shampooing in this study was performed 3 days after the fluralaner spot-on solution treatment, followed by a tick and flea infestation just 1 day later, with an efficacy of 100 % against both parasites. Therefore, water-immersion and shampooing at day 3 following topical administration had no impact on the level of efficacy. Pharmacokinetic data show that fluralaner rapidly crosses the dermis following topical administration and suggest that water-immersion or shampooing will likely not reduce the post-treatment tick and flea efficacy if this occurs earlier than 3 days post-treatment [9].

It is likely that any impact of water-immersion or shampooing on efficacy will be a general effect and will not be specific to any particular ectoparasite species. No negative effect on efficacy of either water-immersion or shampooing was observed for the test parasites chosen (*I. ricinus* or *C. felis*). Consequently, it is expected that there will be no negative impact on the efficacy of any other ectoparasite known to be sensitive to fluralaner.

## Conclusions

Neither water-immersion nor shampooing after single topical administration of fluralaner spot-on solution had an impact on the excellent tick and flea efficacy over the 12-week recommended re-treatment interval.
